# Preoperatively Predicting the Central Lymph Node Metastasis for Papillary Thyroid Cancer Patients With Hashimoto’s Thyroiditis

**DOI:** 10.3389/fendo.2021.713475

**Published:** 2021-07-22

**Authors:** Yu Min, Yizhou Huang, Minjie Wei, Xiaoyuan Wei, Hang Chen, Xing Wang, Jialin Chen, Ke Xiang, Yang Feng, Guobing Yin

**Affiliations:** ^1^ Department of Breast and Thyroid Surgery, The Second Affiliated Hospital of Chongqing Medical University, Chongqing, China; ^2^ Department of Endocrinology, The Second Affiliated Hospital, Chongqing Medical University, Chongqing, China; ^3^ Department of Ultrasound, The Second Affiliated Hospital, Chongqing Medical University, Chongqing, China; ^4^ Department of Cardiology, The Second Affiliated Hospital, Chongqing Medical University, Chongqing, China

**Keywords:** thyroid carcinoma, central lymph node metastasis, ultrasound characteristics, nomogram, risk factor

## Abstract

**Background:**

The preoperative distinguishment of lymph nodes with reactive hyperplasia or tumor metastasis plays a pivotal role in guiding the surgical extension for papillary thyroid carcinoma (PTC) with Hashimoto’s thyroiditis (HT), especially in terms of the central lymph node (CLN) dissection. We aim to identify the preparative risk factors for CLN metastasis in PTC patients concurrent with HT.

**Materials and Methods:**

We retrospectively reviewed and analyzed the data including the basic information, preoperative sonographic characteristics, and thyroid function of consecutive PTC patients with HT in our medical center between Jan 2019 and Apr 2021. The Chi-square and Fisher’s exact tests were used for comparison of qualitative variables among patients with or without CLN metastasis. Univariate and multivariate logistic regression analyses were used to determine the risk factors for CLN metastasis. The nomogram was constructed and further evaluated by two cohorts produced by 1,000 resampling bootstrap analysis.

**Results:**

A total of 98 in 214 (45.8%) PTC patients were identified with CLN metastasis. In multivariate analysis, four variables including high serum thyroglobulin antibody (TgAb) level (>1,150 IU/ml), lower tumor location, irregular margin of CLN, and micro-calcification in the CLN were determined to be significantly associated with the CLN metastasis in PTC patients with HT. An individualized nomogram was consequently established with a favorable C-index of 0.815 and verified *via* two internal validation cohorts.

**Conclusions:**

Our results indicated that preoperatively sonographic characteristics of the tumor and lymph node condition combined with serum TgAb level can significantly predict the CLN in PTC patients with HT and the novel nomogram may further help surgeons to manage the CLN in this subpopulation.

## Introduction

Over the past few years, the standardized clinical management for papillary thyroid carcinoma (PTC) has aroused wide concern in global researchers (1–3), due to the significant increasing prevalence and overdiagnosis of thyroid cancer (4–6). According to the latest management guidelines derived from the American Thyroid Association (ATA), lobectomy without central lymph node dissection (CLND) was evaluated to be a sufficient surgical extension for clinically lymph node negative (cN0) patients with differentiated thyroid carcinoma (DTC) (3). The consensus of “less is more” has been gradually accepted by the thyroid surgeons, which recommends less extensive surgeries, less radioactive iodine, and less surveillance testing (7–9). However, for patients with enlarged cervical lymph nodes at initial screening, whether the prophylactic CLND should be performed was rarely discussed, especially weighing on the surgical completion and the increasing risk of postoperative complications. Notably, with the high concomitant of Hashimoto’s thyroiditis (HT) in PTC (10), reactive hyperplasia was often observed in central lymph nodes (CLN). Moreover, one recent study identified that the number of examined lymph nodes during the CLND was increased in PTC patients with HT. However, it did not increase the detection rate of positive lymph nodes (11). On the other hand, the role of two thyroid antibodies in inducing malignant transformation of the thyroid tissue, regional lymph node invasion, and even the long-term disease-free survival was still unclear (11–14). Thus, how surgeons could preoperatively distinguish malignant from benign lymph nodes in PTC patients coexisting with HT seems to be an interesting topic in the field of thyroid surgery. Recently, several studies (15–18) have evaluated the clinicopathological characteristics associated with CLNM in patients with HT. To the best of our knowledge, none of them measured the ultrasonic characteristics of the CLN in this population.

Hereby, in the present study, we aim to identify the independent clinical risk factors for promoting the CLNM in PTC patients with HT. Besides, an individualized predicting nomogram model was further established to help surgeons make the preoperative clinical decision on whether the prophylactic CLND is warranted.

## Materials and Methods

### Data Source

**Figure 1:**
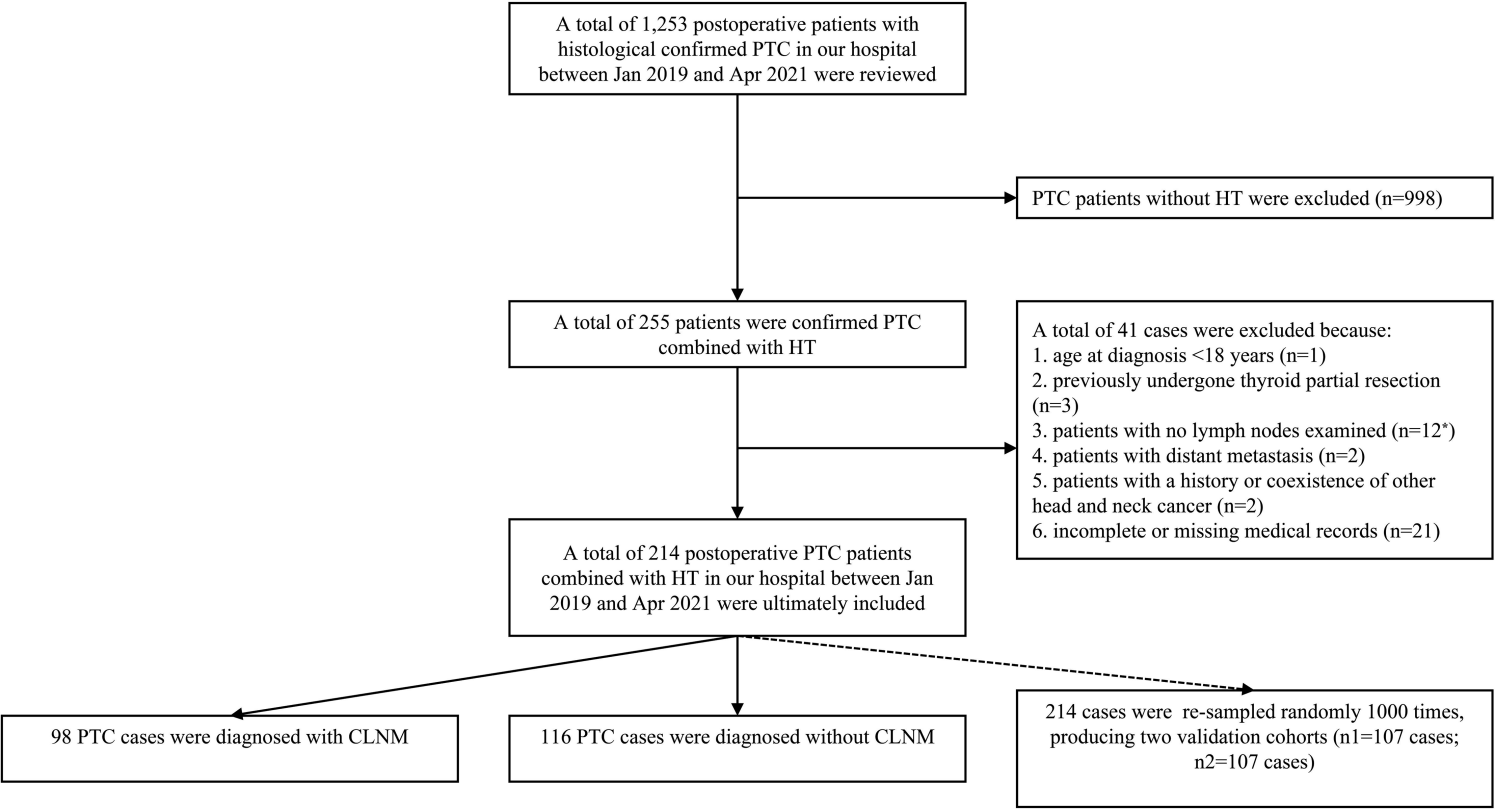
The patient selection process for the present study. Patients with no lymph node examined (n = 12^*^): CLND was not performed or lymph node was not found in the postoperative histopathological examinations.

The data of histological confirmed PTC patients with HT between Jan 2019 and Apr 2021 were derived from the Department of Breast and Thyroid Surgery, the Second Affiliated Hospital of Chongqing Medical University. Ethical approval was waived by the local Ethics Committee of the Chongqing Medical University. The specific including and excluding criteria are summarized in **Figure 1**.

### Variable Definition and Evaluation

The following information was collected to establish a retrospective database:

Basic information: gender (male and female), race (Chinese-Han and Chinese non-Han), age (between 18 and 85 years; divided into two groups: <55 years and ≥55 years according to the 8th edition UICC/AJCC TNM staging system), BRAF^V600E^ mutation status (mutant, wild-type, or not performed), and extrathyroidal invasion (a visible invasion of surrounding muscle or soft tissues by intraoperative findings).

Preoperative Color Doppler ultrasound features: primary tumor size (the largest diameter ≤1 cm and >1 cm), multifocality (more than two primary tumor focus), aspect ratio (anteroposterior diameter/transverse diameter, divided as ≤1 and >1), tumor location (left, right, isthmus, and bilateral of the thyroid gland), tumor longitudinal location (upper, middle, and lower of the thyroid gland), tumor composition (defined as cystic, solid, and solid with cystic), tumor boundary (clear and unclear), margin (smooth, semi-regular, and irregular), calcification (no calcification, microcalcification: <1 mm in diameter, and macrocalcification: >1 mm in diameter; microcalcification is prior to macrocalcification if the tumor presences of both microcalcification and macrocalcification), echogenicity (defined as hypoechoic, isoechoic, and hyperechoic), capsular relation (invasion or not), and blood flow in the tumor (avascularity, peripheral vascularity, limited vascularity, and strip-like vascularity). The central lymph node ultrasound features: lymphadenopathy (yes or not), margin (regular and irregular), lymph Cortex (thinning: the thickness of the cortex is less than half of the diameter of hyperechoic area, normal, thickening: the thickness of the cortex is more than half of the diameter of hyperechoic area), lymphatic hilum (normal, unclear, and disappear), and calcification and blood flow (refer to the tumor calcification and blood flow) (**Table 1**).

**Table 1:** The definitions of the preoperative ultrasound features.

Preoperative ultrasound features	Definition and evaluation
**Tumor**	
primary tumor size	the largest tumor diameter ≤1 cm and >1 cm
Multifocality	more than two primary tumor focuses
aspect ratio	anteroposterior diameter**/**transverse diameter, divided as ≤1 and >1
tumor location	left, right, isthmus, and bilateral of the thyroid gland
tumor longitudinal location	upper, middle, and lower of the thyroid gland
tumor composition	cystic, solid, and solid with cystic
tumor boundary	clear and unclear
margin	smooth, semi-regular, and irregular
^¥^calcification	no calcification, microcalcification, and macrocalcification.
echogenicity	hypoechoic, isoechoic, and hyperechoic
capsular relation	invasion or not
blood flow	avascularity, peripheral vascularity, limited vascularity, and strip-like vascularity
**Central lymph node**	
lymphadenopathy	positive and negative
margin	regular and irregular
^&^lymph cortex	thinning, normal, and thickening
lymphatic hilum	normal, unclear, and disappear
calcification	no calcification, microcalcification, and macrocalcification.
blood flow	avascularity, peripheral vascularity, limited vascularity, and strip-like vascularity

^￥^Calcification: microcalcification: <1 mm in diameter, and macrocalcification: >1 mm in diameter; microcalcification is prior to macrocalcification if the tumor presences of both microcalcification and macrocalcification. ^&^Lymph cortex: thinning: the thickness of the cortex is less than half of the diameter of hyperechoic area, normal, thickening: the thickness of the cortex is more than half of the diameter of hyperechoic area.

Surgical information: All the patients enrolled in the study underwent the lobectomy plus CLND or total thyroidectomy plus CLND. HT condition (HT can be diagnosed as meeting one of the following criteria: i) The ultrasound examination revealed diffuse enlargement of thyroid with abundant blood flow combined with TPOAb >34 IU/L or TgAb >115 IU/L; ii) postoperative histopathological confirmed HT) and the number of CLN harvested and positive. All acquired surgical specimens were examined by two or more board-certified pathologists from the department of pathology of the Second Affiliated Hospital of Chongqing Medical University.

Thyroid function tests feature: TSH (Non-pregnancy reference: 0.35–5.00 μIU/ml), fT3 (reference: 3.10–6.80 pmol/L), fT4 (Non-pregnancy reference: 9.50–24.50 pmol/L), TT3 (reference: 1.00–3.10 nmol/L), TT4 (reference: 60.00–160.00 nmol/L), TgAb (0.00–115.00 IU/ml), TPOAb (0.00–34.00 IU/ml), Tg (reference: 1.40–78.00 μg/L).

### Statistical Analysis

Baseline characteristics by HT were compared using Pearson-chi square test (minimal expected value >5), Fisher’s exact chi-square test (minimal expected value ≤5), and quantitative variables (Student’s two-tailed t-test). Univariate and multivariate logistic regression analyses were used to identify the independent risk factors in patients. A two-tailed P-value of <0.05 was defined as the criterion for variable deletion when performing multivariate analyses. A nomogram for predicting the CLN metastasis based on the results of the multivariate logistic regression analysis was developed and evaluated by two cohorts produced by 1,000 resampling bootstrap analysis and calibration curves as well as decision curve analysis (DCA) curve. All analyses were performed using the SPSS24 (SPSS/IBM, Chicago, IL, USA) and R 2.15.3 software.

## Results

### Clinical Characteristics of PTC Patients With HT

Generally, 214 patients were ultimately enrolled in this study. The CLNM was found in 98 patients (45.8%, 92 female cases and eight male cases). There were significant differences in the tumor size (p = 0.021), tumor location (upper/middle/lower of the thyroid, p <0.001), the margin of the tumor (p = 0.027), calcification in the tumor (p = 0.001), the margin of CLN (p <0.001), the cortex of CLN (p = 0.001), lymphatic hilum (p <0.001), calcification in the CLN (p <0.001), and the extrathyroidal invasion (p = 0.006) among patients with CLNM or not. Notably, regarding the thyroid function test, the mean serum TgAb level in patients with CLNM was remarkably higher than patients without CLNM (656.95 ± 931.09 IU/ml vs. 363.49 ± 440.51 IU/ml, p = 0.002). By contrast, the serum TPOAb level in patients with CLNM was significantly lower than in patients without CLNM (137.70 ± 142.11 IU/ml vs. 195.26 ± 195.13 IU/ml, p = 0.016). Additionally, the CLN examined in the CLNM group was higher than the non-CLNM group (9.28 ± 4.93 vs. 5.80 ± 4.70, p <0.001). Only approximately 23% of patients with CLNM were identified coexisting with lateral lymph node metastasis (LLNM). The specific demographic and clinical characteristics of PTC patients with HT are summarized in **Table 2**.

**Table 2:** Clinicopathological features of 214 PTC patients with HT.

Variables	Subgroup	% of patients		*P*
		With CLNMM (n = 98)	Without CLNM (n = 116)	
**Gender**	male	8	2	^a^ **0.046**
	female	92	98	
**Age**	<55	83	84	^b^0.718
	≥55	17	16	
**Race**	Han	97	96	^a^0.729
	Non-Han	3	4	
**Thyroid nodule**				
**Size**	<1 cm	63	78	^b^ **0.021**
	≥1 cm	37	22	
**BRAF^V600E^ mutation**	No	9	8	^b^0.106
	Yes	83	74	
	N/A	8	18	
**Multifocality**	No	64	72	^b^0.201
	Yes	36	28	
**Aspect ratio**	≤1	60	73	^b^0.683
	>1	40	37	
**Location (L/R/I/B)**	left	42	34	^a^0.357
	right	37	48	
	isthmus	1	2	
	bilateral	20	16	
**Location (U/M/L)**	upper	14	28	^b^ **<0.001**
	middle	38	45	
	lower	48	18	
**Composition**	cystic	0	0	^a^1.000
	solid	98	98	
	solid with cystic	2	2	
**Boundary**	clear	20	32	^b^0.058
	unclear	78	68	
**Margin**	smooth	2	6	^a^ **0.027**
	semi-regular	16	28	
	irregular	82	66	
**Calcification**	No	23	47	^a^ **0.001**
	Micro-calcification	72	50	
	Macro-calcification	4	3	
**Echogenicity**	Hypoechoic	98	95	^a^0.294
	isoechoic	2	5	
	Hyperechoic	0	0	
**Capsular relation**	No	67	78	^b^0.093
	invasion	33	22	
**Blood flow**	avascularity	28	41	^a^0.099
	peripheral vascularity	21	23	
	limited vascularity	46	34	
	strip-like vascularity	5	2	
**Central lymph node**				
**Lymphadenopathy**	No	43	50	^b^0.297
	Yes	57	50	
**Margin**	regular	64	94	^b^ **<0.001**
	irregular	36	6	
**Cortex**	thinning	5	0	^a^ **0.001**
	normal	55	78	
	thickening	40	22	
**Lymphatic hilum**	normal	26	50	^b^ **<0.001**
	unclear	56	44	
	disappear	18	6	
**Calcification**	No	76	97	^b^ **<0.001**
	Micro-calcification	24	3	
	Macro-calcification	0	0	
**Blood flow**	No	31	26	^a^0.347
	limited	66	73	
	strip-like	3	0	
**Thyroid function**				
**TSH**	/	^*^2.79 ± 1.69	3.66 ± 7.17	^c^0.241
**fT3**	/	4.85 ± 1.04	4.62 ± 0.70	^c^0.052
**fT4**	/	17.11 ± 3.51	16.34 ± 2.92	^c^0.077
**tT3**	/	1.73 ± 0.37	1.72 ± 0.38	^c^0.764
**tT4**	/	101.17 ± 22.17	99.82 ± 24.66	^c^0.707
**TgAb**	/	656.95 ± 931.09	363.49 ± 440.51	^c^ **0.002**
**TPOAb**	/	137.70 ± 142.11	195.26 ± 195.13	^c^ **0.016**
**TG**	/	19.55 ± 59.15	32.87 ± 86.98	^c^0.200
**Surgical information**				
	Lobectomy+ CLND	22	33	^/^
	Total thyroidectomy + CLND	78	67	
**Histology**	Classical-variate	94	87	^a^ **0.046**
	Follicular-variate	5	13	
	Other	1	0	
**Extrathyroidal invasion**	No	88	97	^b^ **0.006**
	Yes	12	3	
**CLN examined**	/	9.28 ± 4.93	5.80 ± 4.70	^c^ **<0.001**

PTC, papillary thyroid carcinoma; HT, Hashimoto’s thyroiditis; CLNM, central lymph node metastasis; L/R/I/B, left/right/isthmus/bilateral; U/M/L, upper/middle/low; TSH, thyrotropin; fT3, free triiodothyronine; fT4, free thyroxine; TT3, triiodothyronine; TT4, thyroxine; TgAb, anti-thyroglobulin antibody; TPOAb, anti-thyroid peroxidase antibody; TG, thyroglobulin; CLND, central lymph node dissection; CLN, central lymph node; LLNM, lateral lymph node metastasis.

^*^Mean ± SD.

a Two-tail Fisher exact test.

b Pearson’s Chi-squared test.

c Student’s two-tail t-test.

Bold values indicate statistical significance (p < 0.05).

### Univariate and Multivariate Logistic Regression Analysis of CLN in PTC Patients With HT

A total of 25 selected indicators including gender, age, tumor size, multifocality, BRAF^V600E^ mutation, aspect ratio, tumor location (left/right/isthmus/bilateral), tumor location (upper/middle/lower), tumor boundary, tumor margin, calcification inside the tumor, echogenicity capsular relation, and blood flow of the tumor, and the lymphadenopathy, margin, cortex, lymphatic hilum, calcification, blood flow of the lymph nodes as well as the serum thyroid antibodies and the Tg levels of the PTC patients with HT at the initial screening prior to the thyroidectomy. During the univariate logistic regression analysis, male gender (hazard ratio (HR) = 5.06; 95% confidence intervals (CI): 1.05–24.45, p = 0.043), tumor size >1 cm (HR = 2.01; 95%CI: 1.10–3.66, p = 0.022), lower tumor location (HR = 3.57; 95%CI: 1.65–7.73, p = 0.003), irregular tumor margin (HR = 3.68; 95%CI: 0.74–18.29, p = 0.03), micro-calcification (HR = 2.87; 95%CI:1.57–5.23, p = 0.002), irregular lymph node margin (HR = 8.65; 95%CI: 3.62–20.62, p <0.001), thinning cortex (reference, p=0.002), unclear and no lymphatic hilum (HR = 2.50; 95%CI: 1.36–4.57; HR = 5.96; 95%CI: 2.21–16.07, respectively, p <0.001), micro-calcification CLN (HR = 9.08; 95%CI: 3.02–27.23, p <0.001); serum TgAb level >1,150 IU/ml (HR = 3.74; 95%CI: 1.39–10.04, p = 0.026), and the ratio of TgAb/TPOAb >20 (HR = 3.88; 95%CI: 1.49–10.13, p = 0.021) were significantly associated with the CLNM (**Table 3**). However, only four variables including lower tumor location (HR = 6.85; 95%CI: 2.28–20.54, p = 0.001), irregular margin of the CLN (HR = 6.47; 95%CI: 2.08–20.15, p = 0.001); micro-calcification in CLN (HR = 9.96; 95%CI: 2.344–42.34, p = 0.002), and serum TgAb level >1,150 IU/ml (HR = 7.00; 95%CI: 1.56–31.28, p = 0.007) were the independent risk factors in promoting the CLNM in patients with HT (**Table 4**).

**Table 3:** Univariate logistic regression analysis of 214 PTC patients coexisted with HT for CLNM.

Variables	Subgroup	Univariable	
		Hazard ratio	*P*
**Gender**	female	Reference	**0.043**
	male	5.06 (1.05–24.45)	
**Age**	<55	Reference	0.719
	≥55	1.14 (0.55–2.36)	
**Tumor size (cm)**	≤1	Reference	
	>1	2.01 (1.10–3.66)	**0.022**
**Multifocality**	No	Reference	0.202
	Yes	1.45 (0.81–2.60)	
**BRAF^V600E^ mutation**	No	Reference	0.117
	Yes	0.94 (0.35–2.49)	
	N/A	0.38 (0.11–1.35)	0.683
**Aspect ratio**	≤1	Reference	
	>1	1.12 (0.64–1.95)	
**Location (L/R/I/B)**	left	Reference	0.355
	right	0.61 (0.33–1.12)	
	isthmus	0.47 (0.04–5.45)	
	bilateral	1 (0.46–2.15)	
**Location (U/M/L)**	upper	Reference	**0.003**
	middle	1.67 (0.78–3.56)	
	lower	3.57 (1.65–7.73)	
**Boundary**	clear	Reference	0.060
	unclear	1.82 (0.97–3.42)	
**Margin**	smooth	Reference	**0.030**
	semi-regular	1.69 (0.31–9.11)	
	irregular	3.68 (0.74–18.29)	
**Calcification**	No	Reference	**0.002**
	Micro-calcification	2.87 (1.57–5.23)	
	Macro-calcification	2.34 (0.54–10.20)	
**Echogenicity**	Hypoechoic	Reference	0.245
	isoechoic	0.38 (0.07–1.93)	
**Capsular relation**	No	Reference	0.095
	invasion	1.67 (0.91–3.08)	
**Blood flow**	avascularity	Reference	0.109
	peripheral vascularity	1.35 (0.64–2.84)	
	limited vascularity	1.95 (1.03–3.70)	
	strip-like vascularity	4.35 (0.79–23.98)	
**Central lymph node**			
**Lymphadenopathy**	No	Reference	0.297
	Yes	1.33 (0.77–2.29)	
**Margin**	regular	Reference	**<0.001**
	irregular	8.65 (3.62–20.62)	
**Cortex**	thinning	Reference	**0.002**
	normal	0.12 (0.01–1.05)	
	thickening	0.31 (0.34–2.83)	
**Lymphatic hilum**	normal	Reference	**<0.001**
	unclear	2.50 (1.36–4.57)	
	disappear	5.96 (2.21–16.07)	
**Calcification**	No	Reference	**<0.001**
	micro-calcification	9.08 (3.02–27.23)	
**Blood flow**	avascularity	Reference	0.369
	limited vascularity	0.76 (0.42–1.39)	
	strip-like vascularity	3.00 (0.295–30.49)	
**TSH (μIU/ml)**	≤0.35	Reference	0.816
	>0.35 and ≤5	1.31 (0.35–4.81)	
	>5	1.03 (0.22–4.76)	
**TgAb (IU/ml)**	normal	Reference	**0.026**
	>115 and ≤575	0.94 (0.50–1.76)	
	>575 and ≤1,150	0.74 (0.27–2.02)	
	>1,150	3.74 (1.39–10.04)	
**TPOAb**	normal	Reference	0.070
	>34 and ≤170	1.16 (0.58–2.29)	
	>170 and ≤340	0.97 (0.46–2.07)	
	>340	0.34 (0.13–0.86)	
**TgAb/TPOAb**	≤2	Reference	**0.021**
	>2 and ≤20	1.25 (0.70–2.24)	
	>20	3.88 (1.49–10.13)	

PTC, papillary thyroid carcinoma; HT, Hashimoto’s thyroiditis; CLNM, central lymph node metastasis; L/R/I/B, left/right/isthmus/bilateral; U/M/L, upper/middle/low; TSH, thyrotropin; TgAb, anti-thyroglobulin antibody; TPOAb, anti-thyroid peroxidase antibody.

Bold values indicate statistical significance (p < 0.05).

**Table 4:** Multivariate analysis of 214 PTC patients coexisted with HT and CLNM.

Variables	Subgroup	Multivariable	
		Hazard ratio	*P*
**Gender**	female	Reference	0.106
	male	4.90 (0.71–33.67)	
**Tumor size (cm)**	≤1	Reference	
	>1	1.65 (0.68–4.03)	0.267
**Location (U/M/L)**	upper	Reference	**0.001**
	middle	2.17 (0.74–6.40)	
	lower	6.85 (2.28–20.54)	
**Margin**	smooth	Reference	0.377
	semi-regular	0.95 (0.12–7.09)	
	irregular	1.78 (0.27–11.70)	
**Calcification**	No	Reference	0.280
	Micro-calcification	1.87 (0.85–4.10)	
	Macro-calcification	1.09 (0.13–9.01)	
**Central lymph node**			
**Margin**	regular	Reference	**0.001**
	irregular	6.47 (2.08–20.15)	
**Cortex**	thinning	Reference	0.778
	normal	0.64 (0.05–8.11)	
	thickening	0.85 (0.06–11.27	
**Lymphatic hilum**	normal	Reference	0.144
	unclear	1.70 (0.74–3.88)	
	disappear	3.90 (0.96–15.84)	
**Calcification**	No	Reference	**0.002**
	Micro-calcification	9.96 (2.344–42.34)	
**TgAb (IU/ml)**	normal	Reference	**0.007**
	>115 and ≤575	0.76 (0.31–1.85)	
	>575 and ≤1,150	0.60 (0.14–2.51)	
	>1,150	7.00 (1.56–31.28)	
**TgAb/TPOAb**	≤2	Reference	0.441
	>2 and ≤20	0.85 (0.35–2.09)	
	>20	2.06 (0.47–8.90)	

PTC, papillary thyroid carcinoma; HT, Hashimoto’s thyroiditis; CLNM, central lymph node metastasis; U/M/L, upper/middle/low; TSH, thyrotropin; TgAb, anti-thyroglobulin antibody; TPOAb, anti-thyroid peroxidase antibody; TG, thyroglobulin.

Bold values indicate statistical significance (p < 0.05).

### Nomogram Construction and Validation

**Figure 2:**
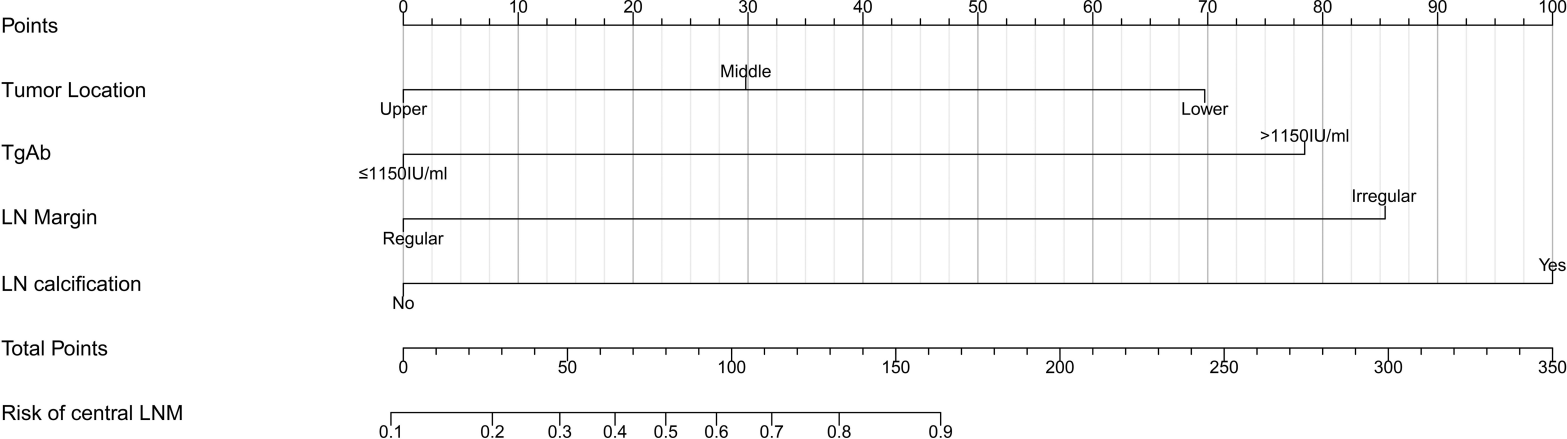
Clinical factor-based nomogram used for preoperatively predicting the CLNM in PTC patients.

**Figure 3:**
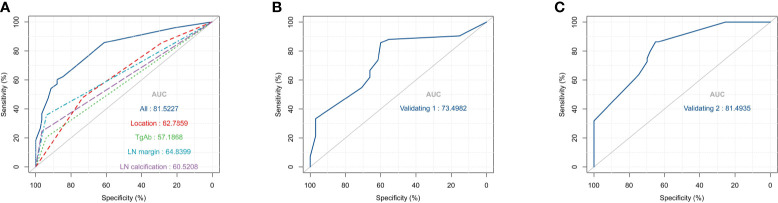
The receiver operating characteristics (ROC) curve and area under the ROC curve (AUC). **(A)** The ROC in the training cohort; **(B)**. The ROC in the first validating cohort; **(C)**. The ROC in the second validating cohort.

**Figure 4:**
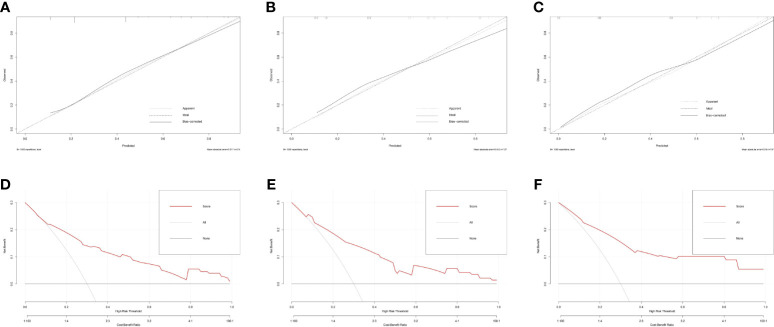
The calibration curves for evaluating the accuracy of the nomogram and determination of decision points *via* Decision Curve Analysis (DCA). **(A)** The calibration curves in the training cohort; **(B)** The calibration curves in the first validation cohort derived from the 1,000 resampling bootstrap analysis; **(C)** The calibration curves in the second validation cohort derived from the 1,000 resampling bootstrap analysis; **(D)** The DCA in training cohort; **(E)** The DCA in the first validation cohort; **(F)** The DCA in the second validation cohort.

Based on the multivariate logistic regression analysis results, four variables including tumor location, serum TgAb level, margin, and calcification of the lymph nodes were used to constructing an intuitive nomogram for predicting the CLNM in patients with HT (**Figure 2**). The specific value of each variable is summarized in **Table S1**. An optimistic C-index (0.815), which was in accordance with the AUC of the ROC (**Figure 3A**), was achieved. The model was further validated by two independent cohorts producing by 1,000 resampling bootstrap analysis which achieved 0.73 (**Figure 3B**) and 0.814 (**Figure 3C**), respectively. To evaluate the utility of the nomogram, three calibration curves of the CLNM risk nomogram in PTC patients with HT were displayed. The curves (apparent, ideal, and bias-corrected lines) suggested a great agreement in the training model as well as two internal validation cohorts (**Table S1**), with a mean absolute error of 0.017 (**Figure 4A**), 0.042 (**Figure 4B**), and 0.03 (**Figure 4C**), respectively. Moreover, the decision curve analyses (DCA) were performed to evaluate the utility of the model in detecting CLNM for PTC patients with HT. The DCA curves presented that the score derived from the nomogram would be more effective than a treat-none or treat-all strategy when the threshold probability ranged from 0.1 to 1.0 in three cohorts (**Figures 4D–F**).

## Discussion

With the increasing prevalence of DTC and the application of thyroid ultrasound around the world, thousands of patients were admitted to the hospital and further elect to undergo surgical intervention (5, 6). However, the trend of overdiagnosis and treatment modality has aroused wide concerns. Researchers were committed to exploring a more proper clinical management guideline to deal with this high prevalence but relatively low mortality disease (1–3, 9). Active surveillance (AS) for low-risk PTMC patients and thyroid lobectomy without prophylactic CLND for PTC patients with cN0 status are gradually accepted as reasonable management strategies, especially in developed countries (7, 8, 19–21). However, in China, the management of CLN has always been a subject of controversy in the field of thyroid surgery (1). Besides, as one of the most frequent autoimmune diseases in thyroid tissue, HT could generally cause cervical lymph node enlargement due to the chronic inflammatory immune response. Additionally, this chronic lymphocytic thyroiditis was often coexisted with the PTC, especially in terms of PTMC (10). As a result, how surgeons could preoperatively distinguish the hyperplastic and malignant lymph nodes becomes a pivotal step in guiding the precision surgical protocol in dealing with the CLN.

Reviewing recent studies on evaluating the prediction risk factors of CLNM in PTC patients (22–24), only a few studies were focusing on the PTC patients with the presence of HT condition (11, 12, 16–18). Moreover, a majority of prediction variables were only dependent on the single examination result, like the thyroid function test (18), sonographic characteristics (16), or clinical factors (17). Although Zhao and colleagues (15) determined multiple preoperative factors in predicting the CLNM in patients with HT, the ultrasound characteristics of CLN seemed to be missed.

In the present study, we included 214 PTC patients with HT to explore the potential risk factors in promoting CLNM based on the combinations of clinical characteristics, thyroid function tests, and ultrasound parameters of these patients. Among 214 PTC patients with HT, 98 of 214 cases (45.8%) were displayed with CLNM, which was similar to the previous reports of Wen et al. (18) and Zhao et al. (15) (44.9 and 45.7%, respectively). It’s believed that preoperative ultrasonography has limited sensitivity for small metastases of PTC, and the accuracy of ultrasound-based diagnosis was highly dependent on radiologists’ experience (22, 25, 26). Similarly, our study showed that the suspected lymphadenopathy was only detected in 57% (55/98 cases) of patients with histological confirmed CLNM during the initial ultrasound examination, which indicated approximately 43% (43/98 cases) of patients with CLNM could be omitted. Thus, univariate and multivariate analyses were performed to discover more indicators in predicting the CLNM. In accordance with the previous findings (16, 24, 27), male gender, tumor size (>1 cm), and micro-calcification of the tumor were the risk factors of CLNM during the univariate analysis. Among the PTC patients with HT, the serum TgAb level was significantly higher in the CLNM group (656.95 ± 931.09 vs. 363.49 ± 440.51, p = 0.002) and the serum TPOAb level was lower in the CLNM group (137.70 ± 142.11 vs. 195.26 ± 195.13, p = 0.016). Furthermore, the multivariate analysis supported that a very high level of serum TgAb, especially 10-fold higher than the reference level (1,150 IU/ml), was an independent risk factor in promoting the CLNM. Although the overall TPOAb level was not determined to be a protective factor in CLNM (p = 0.070), a high level of TPOAb (>340 IU/ml) had a negative correlation with CLNM (HR = 0.34, 95%CI: 0.13–0.86). Interestingly, we identified that the ratio of TgAb and TPOAb levels were significantly associated with the risk of CLNM once the ratio index was higher than 2 (p = 0.021), but this predicting ability was disappeared during the multivariate analysis (p = 0.321). With the same reference standard of thyroid function test, Wen et al. (18) determined that serum TgAb was a risk factor in CLNM as our result presented while Zhao et al. (15) did not find any correlation between antibody statuses and CLNM. On the other hand, the raised level of Tg was frequently used for predicting the recurrence of PTC patients with reasonable sensitivity and specificity during the postoperative follow-up. But some recent studies (28, 29) have proved that serum Tg level was markedly related to distant lymph node metastasis and skip metastasis in PTC patients. However, we did not include this factor in the further analysis as a result of it could be impaired by the HT condition, especially in the presence of TgAb. Besides, we also identified irregular tumor margin, irregular CLN margin, the disappearance of lymphatic hilum, and micro-calcification of the CLN had a positive correlation with the CLNM during the univariate analysis. In addition, regarding the tumor location, it was recently identified to be an indicator for predicting the regional LNM (30, 31). For instance, based on a large Korean population, Back et al. determined that the superior located PTC had a higher risk of lateral lymph node metastasis (adjusted HR = 3.17, p <0.0001) (30). Besides, the results of one recent meta-analysis also yielded that locations of the primary tumor in the central area and low pole were the risk factors of CLNM in cN0 patients (HR = 1.86, p <0.00001) (23). In the present study, we found lower tumor location was significantly associated with CLNM in PTC patients with HT (HR = 6.27, 95%CI: 2.08–18.90; p = 0.002).

After the multivariate analysis, four variables including tumor location (low), high level of TgAb (>1,150 IU/ml), the irregular margin of CLN, and micro-calcification in the CLN were independent risk factors in promoting CLM in patients with HT. However, in PTC patients with HT, we did not successfully validate the clinical factors which were previously confirmed significantly associated with the CLNM like male gender, large tumor size, age <55 years, or multifocality (27, 32, 33). Possible reasons were the relatively small sample size, the different including criteria, and study setting compared with our previous work on predicting the cervical lymph node metastasis in classic PTC patients (34). Thus, the results, especially those that were significant during the univariate analysis but not in multivariate analysis, need to be further evaluated *via* a multicenter study with a larger sample size.

To visualize the results for conveniently applying to the clinical practice, a prediction nomogram was established with four variables involved. Moreover, the C-index of our nomogram was higher than 0.7 and even reached 0.815, which was higher than the C-index of Zhao et al. (15) (0.815 vs. 0.723), indicating that our model has sufficient discrimination ability. Noticeably, the nomogram was further successfully evaluated by two cohorts produced by 1,000 resampling bootstrap analysis. The calibration curves in both training and validation cohorts indicated that the nomogram we developed has a favorable agreement between the ideal and appear lines. Moreover, the DCA graphically shows the clinical usefulness of the model.

Nonetheless, there were still some limitations that should be mentioned. First, the weakness of this nomogram is a lack of external validation which limits the clinical application in other regions. Thus, more external validation cohorts by the prospective study are urgently demanded to further evaluate the feasibility of our nomograms. And the use of intraoperative ultrasound study of central compartment lymph nodes could help surgeons to decide the better surgical approach. Second, this was a retrospective study from a single-center experience which did introduce some selection biases. Third, in our department of pathology of our medical center, whether the lymph nodes were microscopically or macroscopically positive were not further distinguished. Therefore, the future study and validation cohorts are supposed to make up this limitation. Last, there were only four preoperative indicators ultimately included in our nomogram, which indicated there might be potential variables waiting to be discovered that could make our nomogram completer and more reliable in future practice.

## Conclusion

In summary, our results present that lower tumor location, very high serum TgAb level (>1,150 IU/ml), irregular margin, and micro-calcification of the CLN were determined to be significantly associated with the CLNM in PTC patients with HT. The nomogram we constructed maintains satisfying discrimination for preoperatively predicting the risk of CLNM in these patients, which could help surgeons better stratify patients who are at a high-risk level for prophylactic CLND. Meanwhile, we propose more external validations to further strengthen our conclusions.

## Code Availability

The software application generated during and/or analyzed during the current study are available from the corresponding author on reasonable request.

## Data Availability Statement

The raw data supporting the conclusions of this article will be made available by the authors, without undue reservation.

## Ethics Statement

Ethical review and approval was not required for the study on human participants in accordance with the local legislation and institutional requirements. The patients/participants provided their written informed consent to participate in this study.

## Author Contributions

All authors contributed to the conception and design of the study. YM, YF, XWe, and HC organized the database. YM, YF, JC, KX, GY, and MW performed the statistical analysis. All authors wrote the first draft of the manuscript. All authors wrote sections of the manuscript. All authors contributed to the article and approved the submitted version.

## Conflict of Interest

The authors declare that the research was conducted in the absence of any commercial or financial relationships that could be construed as a potential conflict of interest.
