# Supplementary material for: Preoperatively Predicting the Central Lymph Node Metastasis for Papillary Thyroid Cancer Patients With Hashimoto’s Thyroiditis
Source: Front Endocrinol (Lausanne). 2021 Jul 22;12:713475. doi: 10.3389/fendo.2021.713475 (PMC8339927; doi:10.3389/fendo.2021.713475)
Supplement: Supplementary file 1 [file Table_1.docx]

**Table S1**. The specific value of clinical and ultrasound features in the nomogram.

| **Characteristics** | **Score** |
| --- | --- |
| **Tumor location** |  |
| Upper | 0 |
| Middle | 30 |
| Lower | 70 |
| **TgAb level** |  |
| ≤ 1150IU/mL | 0 |
| >1150IU/mL | 78 |
| **CLN margin** |  |
| Regular | 0 |
| Irregular | 85 |
| **CLN calcification** |  |
| No | 0 |
| Micro-calcification | 100 |
| **Total point for predicting the CLNM** |  |
| 0.1 | 0 |
| 0.2 | 27 |
| 0.3 | 48 |
| 0.4 | 64 |
| 0.5 | 80 |
| 0.6 | 95 |
| 0.7 | 112 |
| 0.8 | 133 |
| 0.9 | 164 |

Abbreviation: CLN: central lymph node; CLNM: central lymph node metastasis; TgAb: anti-thyroglobulin antibody.
