# Supplementary material for: Preoperatively Predicting the Central Lymph Node Metastasis for Papillary Thyroid Cancer Patients With Hashimoto’s Thyroiditis
Source: Front Endocrinol (Lausanne). 2021 Jul 22;12:713475. doi: 10.3389/fendo.2021.713475 (PMC8339927; doi:10.3389/fendo.2021.713475)
Supplement: Supplementary file 2 [file Table_2.docx]

Table S2. Clinical and ultrasound features of two validating cohorts obtained from 1000 resampling.

| **Variables** | **Subgroup** | **% of patients** | |
| --- | --- | --- | --- |
|  |  | **Validating cohort 1 (n=107 cases)** | **Validating cohort 2 (n=107 cases)** |
| **Gender** | male | 4 | 4 |
|  | female | 96 | 96 |
| **Age** | <55 | 89 | 84 |
|  | ≥55 | 11 | 16 |
| **Race** | Han | 97 | 96 |
|  | Non-Han | 3 | 4 |
| **Thyroid nodule** |  |  |  |
| **Size** | <1cm | 74 | 69 |
|  | ≥1cm | 26 | 31 |
| **BRAF^V600E^ mutation** | No | 9 | 12 |
|  | Yes | 78 | 77 |
|  | N/A | 13 | 11 |
| **Multifocality** | No | 72 | 76 |
|  | Yes | 28 | 24 |
| **Aspect ratio** | ≤1 | 63 | 64 |
|  | >1 | 37 | 36 |
| **Location (L/R/I/B)** | left | 33 | 35 |
|  | right | 51 | 49 |
|  | isthmus | 0 | 4 |
|  | bilateral | 17 | 12 |
| **Location (U/M/L)** | upper | 25 | 19 |
|  | middle | 38 | 44 |
|  | lower | 37 | 37 |
| **Composition** | cystic | 0 | 0 |
|  | solid | 97 | 98 |
|  | solid with cystic | 3 | 2 |
| **Boundary** | clear | 25 | 29 |
|  | unclear | 75 | 71 |
| **Margin** | smooth | 2 | 7 |
|  | semi-regular | 23 | 20 |
|  | irregular | 75 | 73 |
| **Calcification** | No | 35 | 34 |
|  | Micro-calcification | 61 | 63 |
|  | Macro-calcification | 4 | 3 |
| **Echogenicity** | Hypoechoic | 95 | 93 |
|  | isoechoic | 5 | 7 |
|  | Hyperechoic | 0 | 0 |
| **Capsular relation** | No | 70 | 75 |
|  | invasion | 30 | 25 |
| **Blood flow** | avascularity | 37 | 40 |
|  | peripheral vascularity | 29 | 22 |
|  | limited vascularity | 30 | 36 |
|  | strip-like vascularity | 4 | 2 |
| **Central lymph node** |  |  |  |
| **lymphadenopathy** | No | 36 | 42 |
|  | Yes | 64 | 58 |
| **Margin** | regular | 80 | 86 |
|  | irregular | 20 | 14 |
| **Cortex** | thinning | 3 | 1 |
|  | normal | 68 | 69 |
|  | thickening | 29 | 30 |
| **Lymphatic hilum** | normal | 38 | 48 |
|  | unclear | 49 | 47 |
|  | disappear | 13 | 5 |
| **Calcification** | No | 76 | 97 |
|  | Micro-calcification | 24 | 3 |
|  | Macro-calcification | 0 | 0 |
| **Blood flow** | avascularity | 31 | 23 |
|  | limited vascularity | 67 | 77 |
|  | strip-like vascularity | 2 | 0 |
| ^*^**Thyroid function** |  |  |  |
| **TSH** | / | 2.92±2.02 | 2.62±2.29 |
| **fT3** | / | 4.74±0.87 | 4.69±0.63 |
| **fT4** | / | 16.44±2.94 | 16.75±2.90 |
| **tT3** | / | 1.77±0.41 | 1.71±0.31 |
| **tT4** | / | 102.85±24.54 | 100.50±18.86 |
| **TgAb** | / | 478.33±616.77 | 497.24±766.07 |
| **TPOAb** | / | 156.30±171.59 | 173.89±176.35 |
| **TG** | / | 26.47±76.99 | 30.59±78.20 |
| **Surgical Information** |  |  |  |
|  | Lobectomy+ CLND | 32 | 36 |
|  | Total thyroidectomy +CLND | 68 | 64 |
| **Histology** | Classical-variate | 93 | 90 |
|  | Follicular-variate | 6 | 10 |
|  | Other | 1 | 0 |
| **Extrathyroidal invasion** | No | 95 | 94 |
|  | Yes | 5 | 6 |
| ^*^**CLN examined** | / | 7.42±4.95 | 6.52±5.10 |
| **CLNM** | No | 61 | 59 |
|  | Yes | 39 | 41 |

Abbreviation: PTC: papillary thyroid carcinoma; HT: Hashimoto’s thyroiditis; CLNM: central lymph node metastasis; L/R/I/B: left/right/ isthmus/ bilateral; U/M/L: upper/middle/low; TSH: thyrotropin; fT3: free triiodothyronine fT4: free thyroxine; TT3: triiodothyronine; TT4: thyroxine; TgAb: anti-thyroglobulin antibody; TPOAb: anti-thyroid peroxidase antibody; TG: thyroglobulin; CLN: central lymph node; LLNM: lateral lymph node metastasis.

^*^Mean±SD
